# Neoadjuvant sintilimab and chemotherapy in patients with resectable esophageal squamous cell carcinoma: A prospective, single-arm, phase 2 trial

**DOI:** 10.3389/fimmu.2022.1031171

**Published:** 2022-10-13

**Authors:** Zhi Zhang, Jinjun Ye, Hui Li, Dayong Gu, Mingyu Du, Dashan Ai, Wei Chen, Ying Fang, Xinyu Xu, Chenguang Bai, Kuaile Zhao, Guoren Zhou

**Affiliations:** ^1^ Department of Thoracic Surgery, Affiliated Cancer Hospital of Nanjing Medical University, Jiangsu Cancer Hospital, Jiangsu Institute of Cancer Research, Nanjing, China; ^2^ Department of Radiation Oncology, Affiliated Cancer Hospital of Nanjing Medical University, Jiangsu Cancer Hospital, Jiangsu Institute of Cancer Research, Nanjing, China; ^3^ Department of Radiation Oncology, Affiliated Cancer Hospital of Fudan University, Shanghai, China; ^4^ Department of Oncology, Affiliated Cancer Hospital of Nanjing Medical University, Jiangsu Cancer Hospital, Jiangsu Institute of Cancer Research, Nanjing, China; ^5^ Department of Pathology, Affiliated Cancer Hospital of Nanjing Medical University, Jiangsu Cancer Hospital, Jiangsu Institute of Cancer Research, Nanjing, China; ^6^ Department of Radiology, Affiliated Cancer Hospital of Nanjing Medical University, Jiangsu Cancer Hospital, Jiangsu Institute of Cancer Research, Nanjing, China

**Keywords:** neoadjuvant, sintilimab and chemotherapy, resectable, esophageal squamous cell carcinoma, prospective

## Abstract

**Background:**

Immunotherapy (Programmed cell death 1 blockade) has entered the ranks of advanced esophageal cancer first-line treatment; however, little is known about the efficacy of PD-1 inhibitor as neoadjuvant therapy in resectable esophageal squamous cell carcinoma (ESCC). We aim to evaluate the activity and safety of the neoadjuvant sintilimab combined with chemotherapy in the treatment of resectable thoracic ESCC.

**Methods:**

The enrolled patients with resectable (clinical stage II to IVA) ESCC received neoadjuvant sintilimab injection (200 mg/time, day 1), paclitaxel liposomes (135 mg/m^2^, day 1), and carboplatin (area under curve of 5 mg/mL/min, day 1) every 21 days for 2 cycles, and esophagectomy was performed within 3-6 weeks after the 2 cycles of treatment. The primary endpoint of the study was the pathological complete response (PCR) rate.

**Results:**

From July 2019 to March 2021, a total of 47 patients were enrolled, of which 33 patients (70.2%) had clinical stage III disease. All patients completed the full two-cycle treatment and forty-five patients received radical surgery, including 44 (97.8%) R0 resections. Ten (22.2%) of 45 patients had a PCR, and the major pathological response (MPR) rate was 44.4% (20/45). The grade 3–4 treatment-related adverse events (TRAEs) were mainly neutropenia (6 of 47,12.8%) and leucopenia (8 of 47,17.0%). One (2.1%) patient occurred postoperative immune-associated encephalitis. No delays in surgery were observed.

**Conclusions:**

sintilimab combined with paclitaxel liposome and carboplatin, as demonstrated in this phase II trial to exhibit a relatively high PCR rate and acceptable safety, warrants additional investigation in resectable ESCC.

**Trial Registration:**

http://www.chictr.org.cn/, ChiCTR1900026593.

## Introduction

Worldwide, the number of new cases of esophageal cancer (EC) reached 572,000, and the number of deaths was 509,000 in 2018 (1). In 2015, 246,000 new cases of esophageal cancer were reported in China, making it one of the top 10 common causes of cancer death (2). Esophageal squamous cell carcinoma (ESCC) in China accounts for approximately 90% of esophageal cancer cases (3). Surgery is still the cornerstone of treatment for potentially resectable ESCC. However, among patients with locally advanced EC, the R0 resection rate is low (around 50%), resulting in early recurrence after surgery (4, 5). Preoperative chemotherapy combined surgery was recommended as the standard regimen in Japan (6). Although a moderately high incidence of pathological response after neoadjuvant chemoradiotherapy is reported, the long-term clinical benefit is still suboptimal and unsatisfactory (7–9), which is associated with more postoperative complications and higher postoperative mortality. Preoperative chemotherapy provides the advantages of fewer side effects, ease of tolerance, as well as being easier to administer at general treatment centers. However, compared with preoperative chemoradiotherapy, its effective rate and PCR rate are lower.

The clinical research results of Keynote-590 (10) (enrolled 70% squamous cell carcinoma), CheckMate-648 (11) (enrolled 100% squamous cell carcinoma), and ESCORT-1st (12) (enrolled 100% squamous cell carcinoma) established the important role of immunotherapy combined with chemotherapy in the first-line treatment of advanced esophageal cancer. Sintilimab combined chemotherapy for ESCC significantly prolonged OS and reduced the risk of death by 37.2%, according to the preliminary results of the Research ORIENT-15 (NCT03748134) (13). However, to date, there has been no conclusive evidence to support the effectiveness of neoadjuvant immunotherapy in patients with ESCC.

There is no consensus on the optimal neoadjuvant chemotherapy treatment for patients with resectable locally advanced ESCC. Patients with advanced or locally progressed ESCC have been treated with paclitaxel plus platinum (14, 15), especially in China. Due to the limitation of poor water solubility of paclitaxel, researches on enhancing the tumor targeting of paclitaxel has been carried out continuously (16, 17). When compared to taxol, putting paclitaxel in liposomes results in a higher maximum tolerated dose, better paclitaxel transport into tumor cells, and fewer side effects (18, 19). In China, liposomal paclitaxel was first approved by the State Food and Drug Administration (national medicine permission number: H20030357) in 2003, and its combination with platinum has been utilized to treat advanced ESCC (20–22).

The study mainly observed the efficacy and safety/feasibility of using the combination of neoadjuvant PD-1 blockade with chemotherapy in patients with resectable ESCC, expecting to explore a more effective and less toxic neoadjuvant treatment regimen to improve the clinical outcomes of patients with ESCC.

## Methods

### Study design and participants

This trial was a single-center single-arm, phase II clinical trial performed at the Affiliated Cancer Hospital of Nanjing Medical University. The main eligibility criteria of this study were histologically confirmed, previously untreated esophageal thoracic squamous cell carcinoma, clinical stage II to IVA disease (defined by the eighth edition Union for International Cancer Control) (23), age 18 to 75 years, and Eastern Cooperative Oncology Group (ECOG) performance status of 0 to 1. Moreover, there was no disease progression before enrollment. Patients were excluded if they had esophageal perforation or hematemesis, prior history of autoimmune disease, severe cardiovascular disease, or other concomitant cancers. PD-L1 biomarker expression did not need to be considered in all enrolled patients.

All patients provided written informed consent before enrollment. The study protocol was approved by the clinical research ethics committee of Jiangsu Cancer Hospital. All patients enrolled in this experiment are Chinese.

### Procedures

All patients had tumor clinical assessment, including diagnostic biopsy, esophagography, endoscopic ultrasonography, and boost brain-neck-thorax-abdomen computed tomography and/or positron emission tomography-CT. A routine electrocardiogram, echocardiography, and hematology index-related test were also carried out.

Patients received the following drugs intravenously before undergoing surgical resection (see Research Schematic 1 in **Supplementary File**): sintilimab (200 mg) on day 1 of each 21-day cycle, paclitaxel liposomes (135 mg/m²) on day 1, and carboplatin (area under the curve [AUC] of 5 mg/mL per min) on day 1. To prevent possible anaphylaxis with paclitaxel liposomes, pretreatments were given 30 min before paclitaxel liposome treatment with intravenous dexamethasone, intramuscular injection with a promazine needle, and intravenous drip of cimetidine injection. It should be noted that the interval between dosing should not be less than 20 days and that sintilimab precedes paclitaxel and carboplatin. After two cycles of neoadjuvant immunotherapy combined with chemotherapy, enhanced CT of the neck, chest, and upper abdomen, ultrasound endoscopy, and esophagography were carried out. Two senior radiologists evaluated lymph node response according to Response Evaluation Criteria in Solid Tumors (RECIST, version 1.1) (24), and esophageal lesion response was assessed by esophagography before and after treatment. (For specific evaluation details, see **Supplemental File 1**)

Surgery was scheduled for 21–42 days after the first day of the second treatment cycle. Patients completed radical surgery through the right chest and abdominal incision (Ivor-Lewis method) (25) and underwent two-field lymphadenectomy (the lymph nodes in the middle and lower mediastinum, upper abdomen, and the cervicothoracic junction of patients were selected for dissection). The following pathological evaluation after neoadjuvant therapy referred to the criterion of the College of American Pathologists (CAP)/National Comprehensive Cancer Network (NCCN) (26): All HE slides of patients enrolled in our trial were graded as 0 (PCR, no evidence of vital residual tumor cells), 1 (MPR, 10% or less vital residual tumor cells), 2 (residual cancer foci with interstitial fibrosis), and 3 (few or no tumor cell regression) under the microscope by pathologists.

Toxic effects were assessed according to the National Cancer Institute’s Common Terminology Criteria for Adverse Events (NCI-CTCAE) (27), version 5.0. The specific principles of dosing reduction during treatment are detailed in **Supplemental File 2**.

### Outcomes

The primary endpoints of this study were efficacy (PCR rate as a short-term efficacy surrogate endpoint) and safety/feasibility. Toxicity profiles were assessed according to the NCI-CTCAE (version 5.0) guidelines. The secondary endpoints included disease control rate (DCR), disease-free survival (DFS, calculated from the date of enrollment), CAP/NCCN pathological tumor regression grade (TRG), and overall survival (OS).

### Exploratory analysis

The neutrophil-to-lymphocyte ratio (NLR), lymphocyte-to-monocyte ratio (LMR), platelet-to-lymphocyte ratio (PLR), and systemic immune-inflammation index (SII) have been used to predict therapeutic response in different tumors (28–30). However, few studies have evaluated its efficacy in patients with ESCC who received anti-PD-1 combined with neoadjuvant chemotherapy (31). In this study, the baseline inflammatory indicators of patients were analyzed to observe whether they have certain guiding significance in predicting the pathological efficacy of anti-PD-1 combined with neoadjuvant chemotherapy in the treatment of ESCC.

### Statistical analysis

According to historical literature (32), the pathological complete response rate of neoadjuvant chemotherapy is 6.4%, and 19.2% is expected in our experimental group. The necessary sample size to guarantee an improvement in the PCR rate, with a global alpha risk of 5%, power of 80%, an accrual period of 18 months, and 10% patient loss, was calculated. Results for the primary endpoint were expressed as frequencies and percentages, and the exact two-sided 95% CIs were calculated by use of the Clopper-Pearson method. Survival probabilities were estimated by use of the Kaplan-Meier method. Associations between pathological response to anti-PD-1 plus neoadjuvant chemotherapy and NLR, LMR, PLR, and SII at baseline and post-treatment and their cutoff values were determined by ROC (receiver operating characteristic curve) analysis. SPSS 25.0 and GraphPad Prism 9.1 were used for data analyses.

## Results

### Baseline

**Figure 1:**
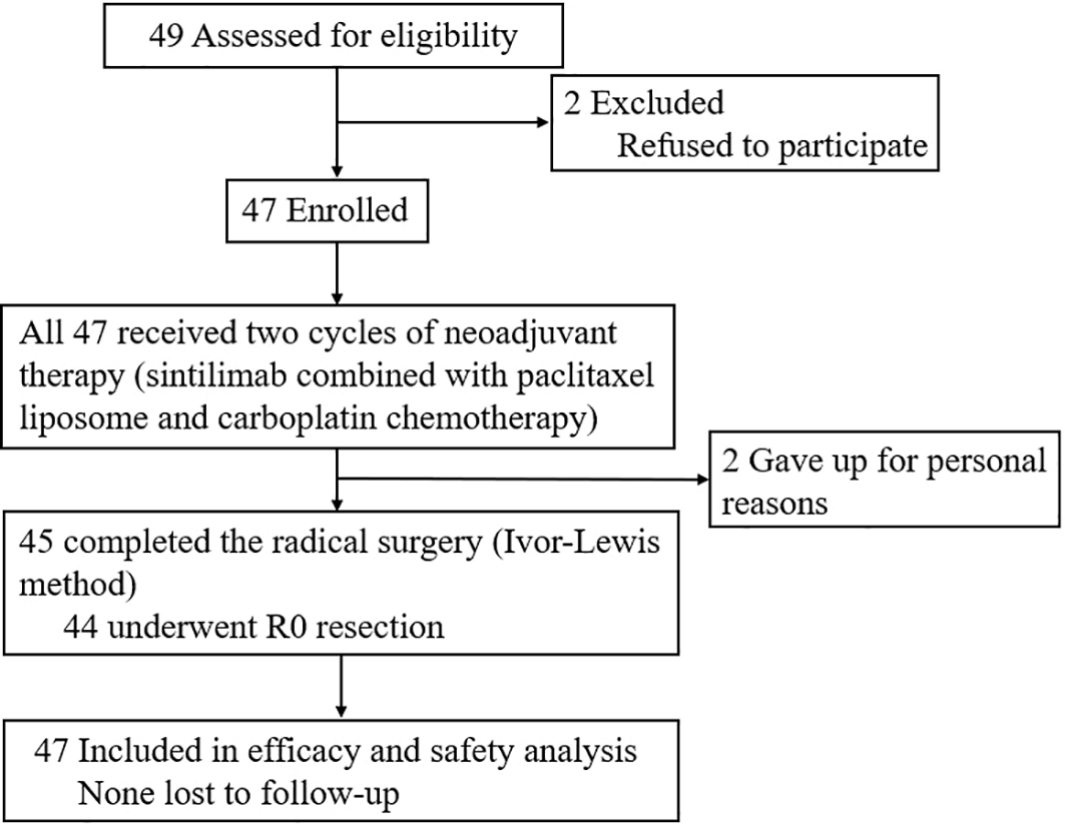
Study flow chart.

From July 2019 to March 2021, 47 patients with esophageal squamous cell carcinoma were enrolled in Jiangsu Tumor Hospital affiliated with Nanjing Medical University, and 45 patients underwent surgery (**Figure 1**). The population included 36 men (76.6%) and 11 women (23.4%). The median age was 66 (IQR, 64-70) years. A total of 38 patients (80.9%) had clinical stage III or IVA disease, and 24 (51.1%) had a tumor length greater than or equal to 5 cm. Nineteen (40.4%) of 47 patients had mid-thoracic esophageal cancer, 25 (53.2%) had lower-thoracic esophageal cancer, and 18 (38.3%) had diabetes, hypertension, or other basic diseases. Other baseline characteristics of the enrolled patients are detailed in **Table 1**.

**Table 1:** Baseline characteristics of enrolled patients (N=47 cases).

Variable	n (%)
Age(y)	Median 66 (IQR,64-70)
<70 ≥70	35 (74.5) 12 (25.5)
Sex	
Male Female	36 (76.6) 11 (23.4)
ECOG performance status	
0	38 (80.9)
1	9 (19.1)
Tumor location	
Middle thoracic Lower thoracic Both	19 (40.4) 25 (53.2) 3 (6.4)
Clinical stage(N)	
cN1	28 (59.6)
cN2	19 (40.6)
Clinical stage (UICC, 8th)	
II III IVA	9 (19.1) 33 (70.2) 5 (10.7)
Tumor length, cm	
<5 ≥5	23 (48.9) 24 (51.1)
Smoking history	
Yes No	28 (59.6) 19 (40.4)
Drinking history	
Yes No	28 (59.6) 19 (40.4)
Medical disease	
Yes No	18 (38.3) 29 (61.7)
Family history of cancer	
Yes No	9 (19.1) 38 (80.9)

UICC, Union for International Cancer Control; ECOG, Eastern Cooperative Oncology Group; IQR, interquartile range.

### Treatment exposure and safety

All 47 enrolled patients completed two cycles of neoadjuvant therapy, and no events of chemotherapy suspension or dose reduction due to physical reasons occurred. TRAEs are summarized in **Table 2**. The most frequently occurring TRAEs of grade 1-2 was anemia, which occurred in 25 (53.2%) of the 47 patients. Leukopenia (20 of 47, 42.6%), hair loss (16 of 47, 34.0%), thrombocytopenia (15 of 47,31.9%), neutropenia (13 of 47, 27.7%), and loss of appetite (12 of 47,25.5%) were also common among the patients. The treatment-related hematological adverse events of grade 3-4 were neutropenia (6[12.8%]), leucopenia (8[17.0%]), anemia (1[2.1%]) and thrombocytopenia (4[8.5%]). One patient developed massive esophageal hemorrhage 3 weeks before surgery but received surgery successfully after positive symptomatic treatment. Immune-related AEs observed during neoadjuvant therapy were all grade 1-2, including rash (4.3%), increased liver transaminases (10.6%), abnormal thyroid function (6.4%), and increased brain natriuretic peptide (14.9%), none of which led to discontinuation of treatment, dose reduction, or surgical delay. In addition, one case of third-degree immune-related encephalitis attributable to neoadjuvant treatment was observed on the 14th postoperative day. The patient suffered from a sudden loss of consciousness and secondary seizures during the postoperative hospital stay. After a comprehensive multidisciplinary discussion, immune-related encephalitis was considered, and glucocorticoid anti-inflammatory therapy and antiepileptic drug therapy were given. As of the last follow-up, the patient’s general condition was stable.

**Table 2:** Adverse Events (N=47 cases).

Adverse event	Grade1-2 (%)	Grade3 (%)	Grade4 (%)
Anemia	25 (53.2)	1 (2.1)	0
Leukopenia	20 (42.6)	6 (12.8)	2 (4.3)
Neutropenia	13 (27.7)	5 (10.6)	1 (2.1)
Thrombocytopenia	15 (31.9)	2 (4.3)	2 (4.3)
Loss of appetite	12 (25.5)	0	0
Nausea	3 (6.4)	0	0
Vomiting	2 (4.3)	0	0
Constipation	3 (6.4)	0	0
Fever	0	0	0
Fatigue	8 (17.0)	0	0
Hair loss	16 (34.0)	0	0
Rash	2 (4.3)	0	0
Increased BNP	7 (14.9)	0	0
Increased ALT	4 (8.5)	0	0
Increased AST	1 (2.1)	0	0
Dizziness	1 (2.1)	0	0
Cardiac toxicity&	1(2.1)	0	0
Hyperthyroidism Encephalitis	3 (6.4) 0	0 1 (2.1)	0 0

BNP, type B natriuretic peptide; ALT, alanine aminotransferase concentrations; AST, aspartate aminotransferase concentrations; &:Increased myocardial enzymes.

### Surgery outcomes

There were no treatment-related surgical delays, but 2 patients gave up for personal reasons, one of whom chose concurrent chemoradiotherapy. The median interval between the last administration of systemic chemotherapy and surgery was 29 days (IQR, 26.5-35 days). Minimally invasive esophagectomy (Ivor-Lewis) was received by 45 patients, of which 44 (97.8%) had a successful R0 resection. The intraoperative blood loss and operative time were 175.0 ± 20.7 mL (mean ± SD) and 228.6 ± 31.1 min, respectively. Surgical complications are reported in **Table 3**. One patient died of hypovolemic shock within 24 hours after surgery. There were three (6.7%) cases of pulmonary infection, one (2.2%) case of anastomotic leakage, and one (2.2%) case of incisional hernia. The median Intensive care unit (ICU) stay was 1 day (range, 0–16) and the median postoperative hospital stay was 13 days (range, 7–52).

**Table 3:** Surgical outcomes (N=45 cases).

Characteristics	n/N (%) or mean ± SD or median (range)
Margins	
Negative Positive	44/45 (97.8%) 1/45 (2.2%)
Changes in lymph node staging status	
Downstaging Unchanged staging Upstaging Blood loss (mL) Cumulative operative time (min) Postoperative hospital stay (day) ICU stay (day)	39/45 (86.7%) 4/45 (8.9%) 2/45 (4.4%) 175.0 ± 20.7 228.6 ± 31.1 13 (7–52) 1 (0–16)
Surgical complications	
Anastomotic leakage Pulmonary infection incisional hernia In- hospital mortality$ 30- day mortality 90- day mortality	1/45 (2.2%) 3/45 (6.7%) 1/45 (2.2%) 1/45 (2.2%) 0 0

Only patients who had undergone surgical treatment were counted; ICU, intensive care unit; $ One patient died of hypovolemic shock within 24 hours after surgery.

### Efficacy

**Figure 2:**
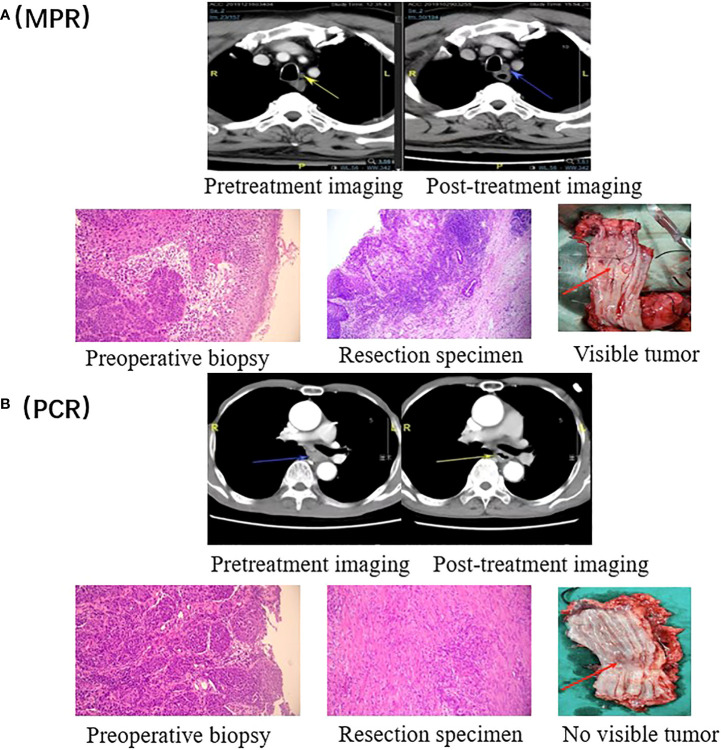
Radiographic and pathological responses. **(A)** Pretreatment and post- treatment CT and H&E images of a representative patient with a pathological response of MPR. The tumor is visible in the resected esophagus. **(B)** Pretreatment and post- treatment CT and H&E images of a representative patient with a PCR. There is no tumor visible in the resected esophagus.

**Figure 3:**
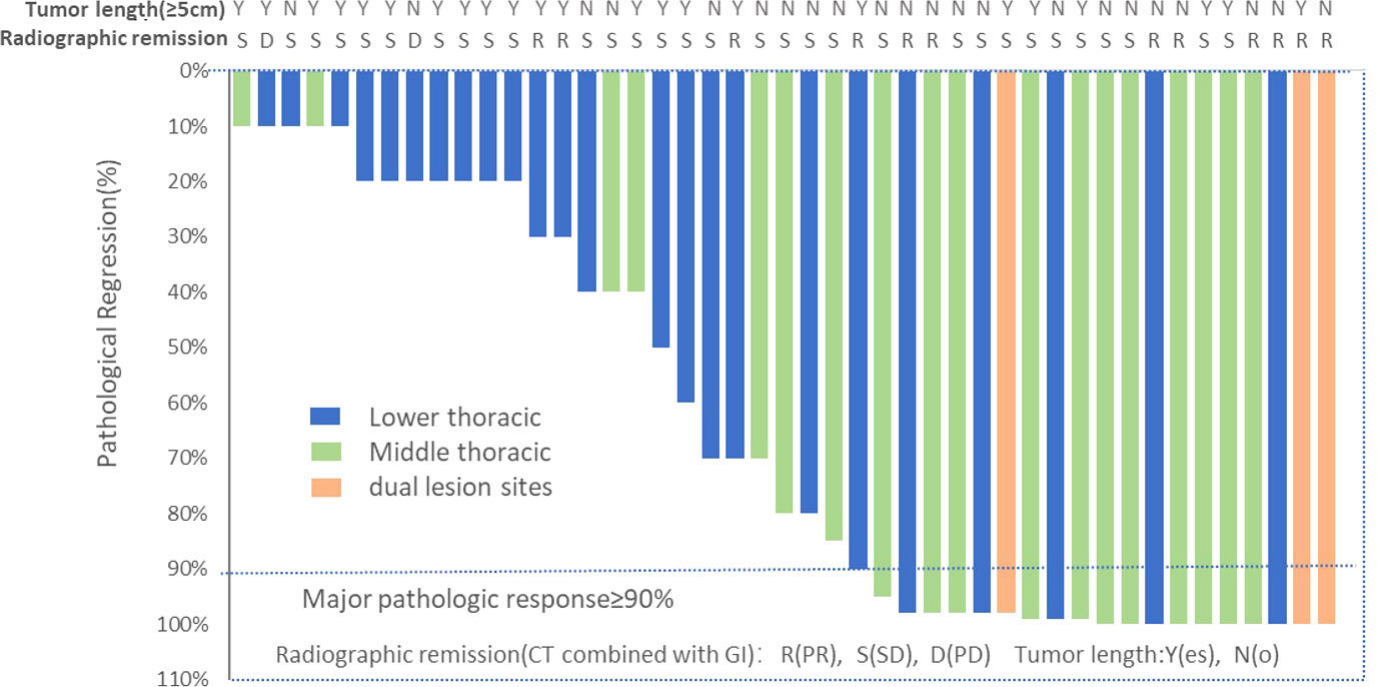
Waterfall plot of pathological tumor regression in the population (N=45). Each bar represents one patient. The upper column shows clinical characteristics and radiological responses.

Ten (22.2%) of 45 patients who underwent successful surgical resection achieved a pathological complete response, and a major pathological response was observed in 20 (44.4%) patients. We also assessed the relationship between patient baseline characteristics and tumor pathological response in a *post hoc* analysis (**Supplemental File 3**). Of 18 patients with mid-thoracic esophageal squamous cell carcinoma who underwent successful surgical resection, 11 (61.1%) patients had a major pathological response, including 6 (33.3%) patients with pathological complete response. (**Figure 2**) In contrast, of 24 patients with lower-thoracic esophageal squamous cell carcinoma who underwent successful surgical resection, 6 (25.0%) patients had a major pathological response, and 2 (8.3%) had a pathological complete response (p=0.02). The distribution of the pathological response of primary tumor can be seen more visually through the waterfall plot (**Figure 3**). The median number of lymph nodes resected was 16 (IQR, 13-21). Among all patients who received surgery, 39 (86.7%) patients achieved pathological downstaging of clinical N stage, including 34 (75.6%) patients with decreased postoperative lymph node staging to pN0 and 5 (11.1%) patients with decreased postoperative lymph node staging to pN1. Lymph node staging remained unchanged in 4 (8.9%) patients, and lymph node progression occurred in 2 (4.4%) patients (both from cN1 to pN3). Of the 32 patients with pathologically confirmed N1/2 stage disease at baseline, 20 (62.5%) had nodal clearing after neoadjuvant treatment (downstaging from cN1/2 to pN0).

According to the comprehensive evaluation, after two cycles of neoadjuvant immunotherapy combined with chemotherapy, 12 (25.5%) patients were assessed as having a partial response, 33 (70.2%) patients achieved stable condition, and 2 (4.3%) patients had disease progression (no distant metastasis occurred). All patients were evaluated and deemed eligible for surgical treatment.

### Follow-up

At the time of analysis (November 2021), the surviving patients had a median follow-up of 14.6 months (IQR, 11.3-24.0 months). No patient was lost to follow-up. A total of 4 (8.5%) patients died, three (6.4%) died of tumor cause and the other one (2.1%) died of postoperative hypovolemic shock. A total of 15 (31.9%) patients suffered recurrence. Nine (19.1%) had regional recurrence only and there were 6 patients (12.8%) with distant metastasis only, consisting of 2 (4.3%) patients with liver metastasis, 1 (2.1%) patient with abdominal metastasis, 1 (2.1%) patient with kidney metastasis, 1 (2.1%) patient with lung metastasis, and 1 (2.1%) patient with brain metastasis.

**Figure 4:**
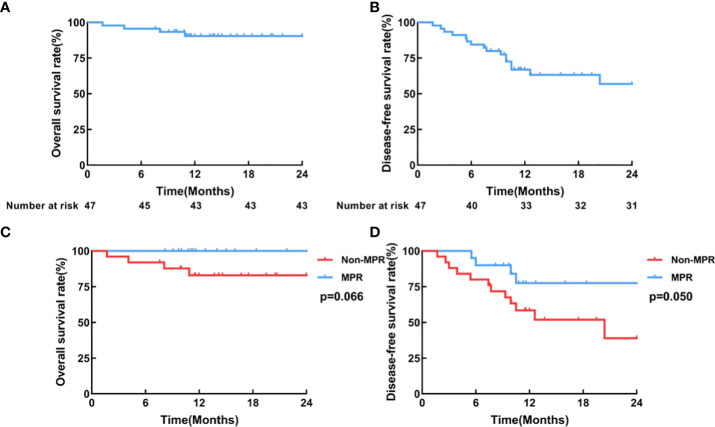
Survival curves. **(A)** Overall survival, **(B)** Disease-free survival curve of all patients who received surgery (N=45); **(C)** Overall survival, **(D)** Disease-free survival curves of the MPR group (n=20) and the non-MPR group (n=25).

In the entire patient cohort, the median disease-free survival (DFS) and the median overall survival (OS) were not reached (**Figures 4A, B**). The 1-year OS was 90.8%, and the 1-year DFS was 68.3%. In *post hoc* analyses of survival, we found that patients who achieved MPR had significantly improved DFS (P=0.050; HR=0.35, 95%CI=0.13-0.92) and OS (P=0.066; HR=0.16, 95%CI=0.02-1.13), compared with those who did not. (**Figures 4C, D**)

### Exploratory analysis

**Figure 5:**
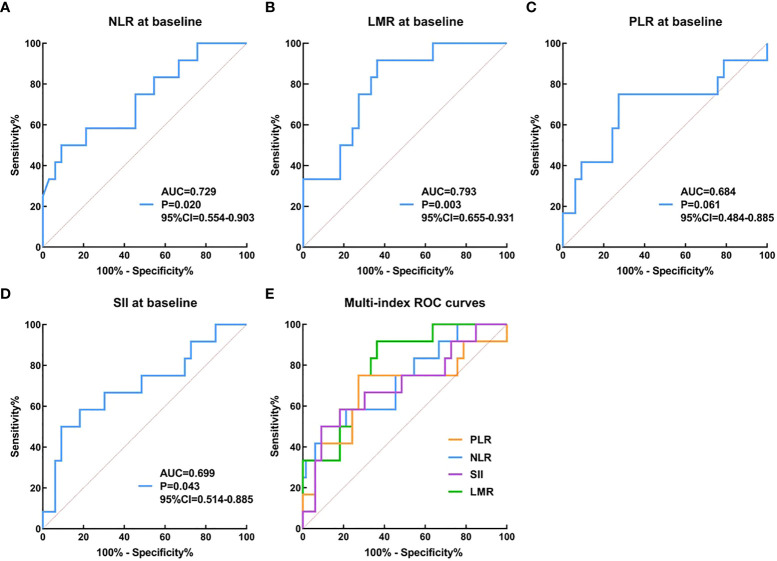
The prediction ability of serum inflammation indexes to distinguish pathological efficacy. **(A–E)**: the therapeutic efficacy was categorized into pathological tumor regression grades 0, 1, and 2 (response) and grade 3 (no response or poor response).

When the therapeutic efficacy of patients with anti-PD-1 plus chemotherapy was divided into CAP/NCCN pathological tumor regression grade 0 (PCR) and grade 1, 2, 3 (non-PCR), our results seemed to show baseline NLR、LMR、PLR、SII could not better predict the pathological tumor regression grade by ROC curve analysis. When the therapeutic efficacy was categorized into pathological tumor regression grades 0, 1, and 2 (response) and grade 3 (no response or poor response), ROC curve analysis showed that NLR at baseline (cutoff=3.29, AUC=0.729, 95% CI 0.554–0.903, P= 0.020, sensitivity=0.50, specificity=0.91, **Figure 5A**), LMR at baseline (cutoff=3.57, AUC= 0.793, 95% CI 0.655–0.931, P=0.003, sensitivity=0.64, specificity=0.92, **Figure 5B**), PLR at baseline (cutoff=143.23, AUC=0.684, 95% CI 0.484–0.885, P=0.061, sensitivity=0.75, specificity=0.73, **Figure 5C**) and SII at baseline (cutoff=815.50, AUC=0.699, 95% CI 0.514–0.885, P=0.043, sensitivity=0.50, specificity=0.91, **Figure 5D**) could be used to predict pathological tumor regression grade. Besides, our results indicated a good predictive performance for MPR involving LMR at baseline (**Supplemental File 4**).

## Discussion

This study prospectively observed the efficacy and safety of radical surgery after neoadjuvant PD1 (sintilimab) combined with chemotherapy in operable esophageal squamous cell carcinoma. To our knowledge, there is no relevant large-sample prospective study at home or abroad, so this trial can explore a new model for the clinical treatment of potentially resectable esophageal squamous cell carcinoma.

Based on the findings of several landmark studies (CROSS study, NEOCRTEC5010, and CheckMate-577 trial) (33–35), neoadjuvant chemoradiotherapy (NCRT) plus surgery has become a recommended treatment option for locally advanced ESCC, especially in most western countries. However, the improved PCR rate in NCRT failed to provide a more significant long-term survival benefit than in NCT (7–9). In addition, the clinical application of NCRT is restricted due to the superimposed toxicity of chemotherapy and radiotherapy. Dose reduction of chemoradiotherapy due to high toxicity weakens the patients’ treatment adherence to a certain extent. In addition, NCRT may further add to the difficulty of surgical procedures (e.g., tissue adhesion and oedema) and increase perioperative complications (e.g., respiratory failure caused by radiation pneumonitis), which undesirably counteracts the survival benefits expected from NCRT.

In terms of toxicity, the incidence of the treatment-related hematological adverse events of grade 3-4 in this study was 40.4%, which was lower than that reported in the NEOCRTEC5010 neoadjuvant chemoradiotherapy group (54.3%) (33). Except for one case of immune-related encephalitis, all immune-related AEs were grade 1. In terms of surgical safety, the neoadjuvant therapy in this study did not delay surgery and the R0 resection rate reached 98%, while in previous studies the reported R0 resection rates with neoadjuvant chemotherapy and neoadjuvant chemoradiotherapy were 60% and 98% (5, 33). The mean number of lymph nodes resected (16.0) and that reported in the CROSS (15.0) study was similar (35). These results suggest that this neoadjuvant therapy can result in high R0 resection rates, greatly reducing the difficulty for surgeons to completely remove the primary tumor or lymph nodes. In the aspect of postoperative complications, the incidence of anastomotic fistula in our study was 2.2%, which was lower than that previously reported in the CROSS study (22%) (35). Although there was one perioperative death, it was deemed unrelated to neoadjuvant therapy. In general, neoadjuvant chemotherapy combined with immunotherapy was well tolerated and safe.

Encouragingly, in this study, the PCR rate of neoadjuvant therapy with sintilimab combined with carboplatin and paclitaxel liposome reached 22.2%, which was higher than that of previously reported neoadjuvant chemotherapy (6.4%) (32) and similar to the two previous studies of neoadjuvant PD-1 blockade combined with chemotherapy (33%, 25%) (36, 37). We were pleasantly surprised to find that ESCC patients located in the mid-thoracic segment were associated with a more significant pathological response rate, which may be related to the shorter lesion length and lower lymph node stage at baseline in these patients compared with lower segment ESCC patients.We mainly consider the following reasons for the difference in PCR rate between this study and the CROSS study: 1), the addition of radiotherapy in the CROSS study brought better local control; 2), the patients enrolled in the CROSS study had a relatively early tumor stage (stage II or III), meanwhile, 11% of stage IVA patients were included in our study. It was also found in our study that obtaining MPR after neoadjuvant therapy was associated with better survival outcomes. However, whether this could translate into long-term survival benefits requires further research.

In ESCC studies, meta-analysis showed that clinical indicators such as NLR, PLR, LMR, and SII had moderate predictive value for prognosis (38), yet their potential prediction ability of therapeutic efficacy, especially in connection to immunotherapy, remains rarely documented. In our *post hoc* exploratory analysis, we found that serum inflammatory indexes at baseline in patients appeared to be predictors of pathological response. The model we constructed is easily applicable for clinical practice at no additional cost. Further verification is required to assess whether combining these inflammatory markers results in better predictive performance.

The significance of PD-L1 expression level in tumor immunotherapy has always been a research hotspot. According to the newly published ORIENT-15 study results (13), regardless of the level of PD-L1 expression, sintilimab combined with chemotherapy has benefits in the whole population, including the population with negative PD-L1 expression, so PD-L1 detection is considered non-essential. In addition, according to previous studies on the use of PD1 inhibitors in neoadjuvant therapy for esophageal squamous cell carcinoma, there was no significant correlation between PD-L1 expression and pathological response (39, 40), so the determination of PD-L1 level in tumor tissue was not mandatory in our study design. However, the guiding value of PD-L1 expression level in immunotherapy has always been recognized, and it is worthy of further exploration in subsequent large-sample studies.

### Limitations

There are some limitations to this study. First, because of this study being an exploratory pilot study, the number of enrolled patients was small and Interfering factors have a significant impact. Therefore, our findings and the survival data need to be interpreted with caution. Second, the follow-up time was short and longer follow-ups are needed to assess whether neoadjuvant immunochemotherapy can provide long-term survival benefits for patients. Third, indeed, as a taxane drug, paclitaxel liposome has its advantages, but due to its limited availability, the application of the results derived from this study to other parts of the world requires caution. Further investigation into the optimal duration of treatment and predictor of pathological response should be given more attention.

## Conclusions

In general, for patients with operable esophageal squamous cell carcinoma, neoadjuvant sintilimab combined with chemotherapy followed by radical surgery is feasible and safe. With a high proportion of patients obtaining a pathological complete response, this regimen has favorable antitumor efficacy and is worthy of further test in a large sample prospective study.

## Data availability statement

The original contributions presented in the study are included in the article/**Supplementary Material**. Further inquiries can be directed to the corresponding authors.

## Ethics statement

The studies involving human participants were reviewed and approved by Ethics Committee of Jiangsu Cancer Hospital. The patients/participants provided their written informed consent to participate in this study.

## Author contributions

GZ, KZ, ZZ, JY, and HL had full access to all the data in the study and take responsibility for the integrity of the data and the accuracy of the data analysis. Concept and design: JY, ZZ, GZ, and KZ. Acquisition, analysis, or interpretation of data: HL, JY, DA. Drafting of the manuscript: HL, JY, ZZ. Critical revision of the manuscript for important intellectual content: JY, ZZ, HL, GZ, KZ, DG, MD, DA, WC, YF, XX, CB. All authors contributed to the article and approved the submitted version.

## Funding

This work was supported by Six Talent Peaks Project in Jiangsu Province (CN) [grant numbers: TD-SWYY-007]; Wu Jieping Medical Foundation Project (CN) [grant numbers: 320.6750.19194-60].

## Conflict of interest

The authors declare that the research was conducted in the absence of any commercial or financial relationships that could be construed as a potential conflict of interest.

## Publisher’s note

All claims expressed in this article are solely those of the authors and do not necessarily represent those of their affiliated organizations, or those of the publisher, the editors and the reviewers. Any product that may be evaluated in this article, or claim that may be made by its manufacturer, is not guaranteed or endorsed by the publisher.
