# Supplementary material for: Neoadjuvant sintilimab and chemotherapy in patients with resectable esophageal squamous cell carcinoma: A prospective, single-arm, phase 2 trial
Source: Front Immunol. 2022 Oct 13;13:1031171. doi: 10.3389/fimmu.2022.1031171 (PMC9606329; doi:10.3389/fimmu.2022.1031171)
Supplement: Supplementary file 1 [file DataSheet_1.pdf]

Research Schematic 1

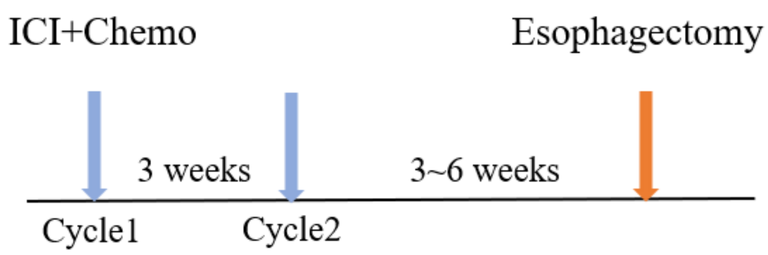

ICI+Chemo: immune checkpoint inhibitor+chemotherapy

supplemental file 1

Specific radiological response assessment details

(a). Mediastinal lymph node (Measurable lesion)

Evaluation standard (RECIST)

Comparison with baseline chest CT:

Complete Response (CR)	All lesions disappeared.
Partial Remission (PR)	The sum of the longest diameter of lesions decreased by more than or equal to 30%.
Stable Disease (SD)	The sum of the longest diameter of lesions does not meet the evaluation criteria of disease progression and partial remission.
Progressive Disease (PD)	The sum of the longest diameter of lesions increased by 20% or more.

(b). Esophageal lesion (Unmeasurable lesion)

Evaluation standard (Modified from "Efficacy Evaluation Criteria of Esophageal Barium Swallowing Radiography at the End of Radiotherapy for Esophageal Cancer in Japan")

Complete Response (CR)	The lesion disappeared completely, the esophageal wall was soft, and no tumor remained.
Partial Remission (PR)	Along the long axis of the esophagus, the length of the lesion subsided by more than 50%, but not completely, and the esophageal lumen stenosis improved.
Stable Disease (SD)	In the long axis of the esophagus, the length of the lesion was less than 50%, or regardless of tumor changes, lumen stenosis of the esophagus is evident but does not progress.

Progressive Disease (PD)	The length of lesions increased by more than 20%, and esophageal lumen stenosis was more obvious than before.

(c). Comprehensive evaluation

lymph node / esophageal lesion	CR	PR	SD	PD
CR	CR	PR	SD	PD
PR	PR	PR	SD	PD
SD	SD	SD	SD	PD
PD	PD	PD	PD	PD

supplemental file 2

Dose adjustment principle

All patients were given the planned chemotherapy dose in principle, and the dose could be adjusted if necessary according to the most serious hematological or other toxicity. Any patient requiring a dose reduction will continue to receive the reduced dose for subsequent treatment cycles. If a patient presents with multiple toxicities and the principles of dose adjustment differ from one another, the lowest dose is selected. If ANC ≤ 1.5 x 109/L and platelets <100 x 109/L on day 1 of the course of treatment, the medication was suspended for one week. Except for alopecia, nausea and vomiting, other non-hematological toxicities of grade II and above should be recovered to grade 0 to 1 before the next course of treatment can be performed. Recovery of hematologic and non-hematologic toxicities can be delayed up to 14 days. A maximum of two decrements are allowed. Dose reductions should be based on the grade of the most severe toxicity from the previous course of treatment. Cisplatin and carboplatin were adjusted to 75% of the original dose for the first time, and to 50% of the original dose for the second time. Paclitaxel was adjusted to 80% of the original dose for the first time, and adjusted to 60% of the original dose for the second time, and the dose adjustment scheme was adjusted according to the following.

1. Paclitaxel and DDP were both reduced: Grade III/IV neutropenia accompanied (≥38.3°C or 38.0°C for 1 hour). Grade IV neutropenia persisting for ≥7 days; other investigators believe that dose reduction is warranted.
2. Cisplatin dose reduction: Adjust the dose of DDP according to the renal function. When 45ml/min≤ creatinine clearance (CCI) <60ml/min, the DDP dose is reduced by 25%; when 30ml/min ≤ creatinine clearance (CCI) <45ml/min, the DDP dose is reduced by 50%; when creatinine clearance (CCI) <30ml/min, the medication should be suspended. The next cycle of treatment cannot be started until recovery to level 0-1.
3. Paclitaxel dose reduction: For severe peripheral neuropathy, the dose should be adjusted or discontinued according to the total dose principle.

Note: DDP is only decremented once when both 1 and 2 are present; Paclitaxel was

reduced only once when both 1 and 3 occurred.

supplemental file 3

The relationship between patient baseline characteristics and tumor pathological response

	MPR（%）	PCR（%）
Had successful surgical resection	20/45（44.4）	10/45（22.2）
Tumor location*		
Middle thoracic	11/18（61.1）	6/18（33.3）
Lower thoracic	6/24（25.0）	2/24（8.3）
P value	0.02	0.02
Tumor length, cm		
＜5	13/22（59.1）	7/22（31.8）
⩾5	7/23（30.4）	3/23（13.0）
P value	0.05	0.12

Abbreviations: Only includes patients who underwent surgical resection. *Three patients with dual lesion sites were not included.

supplemental file 4

ROC curves of serum inflammatory indexes predicting ability of pathological curative effect

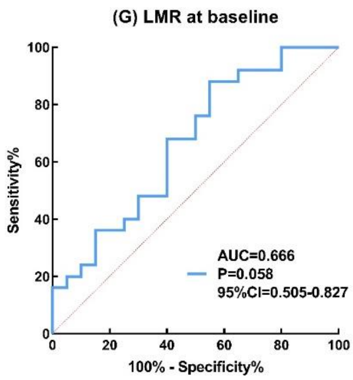

(G): the therapeutic efficacy was categorized into pathological tumor regression grades 0, 1 (MPR) and grade 2, 3 (non-MPR).

Cutoff = 4.19, Sensitivity = 0.45, Specificity = 0.88
